# Vertebral Bone Marrow-Derived Mesenchymal Stromal Cells from Osteoporotic and Healthy Patients Possess Similar Differentiation Properties In Vitro

**DOI:** 10.3390/ijms21218309

**Published:** 2020-11-05

**Authors:** El-Mustapha Haddouti, Thomas M. Randau, Cäcilia Hilgers, Werner Masson, Robert Pflugmacher, Christof Burger, Sascha Gravius, Frank A. Schildberg

**Affiliations:** 1 Clinic for Orthopedics and Trauma Surgery, University Hospital Bonn, 53127 Bonn, Germany; El-Mustapha.Haddouti@ukbonn.de (E.-M.H.); thomas.randau@ukbonn.de (T.M.R.); caecilia.hilgers@ukbonn.de (C.H.); werner.masson@ukbonn.de (W.M.); robert.pflugmacher@ukbonn.de (R.P.); christof.burger@ukbonn.de (C.B.); Sascha.Gravius@umm.de (S.G.); 2 Department of Orthopaedics and Trauma Surgery, University Medical Center Mannheim of University Heidelberg, 68167 Mannheim, Germany

**Keywords:** mesenchymal stromal cells, osteoporosis, immunomodulation, differentiation, proliferation rate, surface markers

## Abstract

Osteoporosis is a disease characterized by low bone mass and an increased risk of fractures. Although several cellular players leading to osteoporosis have been identified, the role of mesenchymal stromal cells (MSC) is still not fully elaborated. The aim of this study was, therefore, to isolate and characterize MSCs from vertebral body of healthy non-osteoporotic and osteoporotic patients, with a particular focus on their osteogenic differentiation potential. Isolated MSCs were characterized by their osteogenic, adipogenic, and chondrogenic differentiation, as well as surface marker expression, proliferation behavior, and immunomodulatory capacity. The mineralization process was confirmed using Alizarin Red S and alkaline phosphatase (ALP) stains and further evaluated by determining ALP activity, mineral deposition, and free phosphate ion release. MSCs from both healthy and osteoporotic patients showed common fibroblast-like morphology and similar proliferation behavior. They expressed the typical MSC surface markers and possessed immunomodulatory capacity. Both groups demonstrated solid trilineage differentiation potential; osteogenic differentiation was further confirmed by increased ALP activity, deposition of inorganic crystals, phosphate ion release, and expression of osteoblast marker genes. Overall, MSCs from osteoporotic and non-osteoporotic patients showed neither a difference in general MSC features nor in the detailed analysis regarding osteogenic differentiation. These data suggest that vertebral body MSCs from osteoporotic patients were not impaired; rather, they possessed full osteogenic potential compared to MSCs from non-osteoporotic patients.

## 1. Introduction

Osteoporosis is a common skeletal disease that is defined by a systemic deterioration of bone mass and increased fragility. Unfortunately, current therapies are still not satisfactory, with osteoporosis increasingly recognized as a major public health issue [1]. Present treatments of osteoporosis are mostly focused on preventing bone resorption and sustaining bone density, but unfortunately, also cause serious side effects [2]. There is, therefore, an urgent need for alternative innovative therapies that promote continuous bone sustainability and regeneration in patients with osteoporosis.

In general, osteoporosis is the consequence of the dysregulation between bone resorption and new bone formation [3,4], which is mediated by osteoblast and osteoclast cell lineages [5,6]. Growing evidence has indicated that bone marrow-derived mesenchymal stromal cells (BMSC), the progenitors of osteoblasts, play a crucial role in osteoporosis [7]. These cells are multipotent, and under physiological conditions, their precisely adjusted osteogenic and adipogenic properties contribute to bone tissue homeostasis [8]. However, several factors, such as menopause or aging, perturb this homeostatic equilibrium, eventually leading to a disbalanced production of bone marrow adipocytes and bone mass loss [9].

Recent publications have shown that the osteogenic potential of mesenchymal stromal cells (MSC) is significantly altered in osteoporosis [10]. Specifically, MSCs from osteoporotic patients possessed a lower ability to differentiate into osteoblasts, as well as displaying a lower growth rate compared to cells from healthy patients [11,12]. Most of these studies, however, did not consider the source of MSCs. This is of particular importance as recent studies could show that tissue source and harvesting technique have a great impact on MSC performance, which is clearly underestimated in the present literature and requires further investigation [13,14,15,16].

Currently, there are many reports considering MSC-based therapy for osteoporosis as a novel approach to overcome the limitations of the present treatments [2,17,18,19]. Different tissue sources for stromal cell-based therapy for osteoporosis, including bone marrow, adipose tissue, perinatal-derived MSCs, as well as small molecules for endogenous stromal cell recruitment, have been suggested [2,18,19,20,21]. Some of these led to a number of preclinical studies testing MSC transplantation in small animal models for osteoporosis; however, these studies were met with divergent outcomes [17,19]. An explanation for these inconsistent results may be the already mentioned lack of standardized protocols for MSC isolation, expansion, and characterization, as well as the use of different tissue sources and species. These parameters significantly influence MSC phenotype and functionality [13,14,15,16,22]. The present literature shows that we are still at the beginning of decoding MSC features because of their heterogeneity and that a more detailed analysis of their complex biology is needed to understand better how they can be used in a clinical setting [23].

One important MSC niche in the context of osteoporosis is the spine. It is commonly affected by osteoporosis, which is also evident from vertebral compression fractures, which are a frequent occurrence in osteoporotic patients and heal poorly [24,25]. Although several animal models of osteoporosis are available, experimental animals, such as ovariectomized rats or sheep, develop osteoporosis that is not fully consistent with the pathogenesis of human osteoporosis [26]. Unfortunately, only comparatively few examinations of the human vertebral body as a source for MSCs have hitherto been carried out because of its anatomically delicate position, which makes it a great deal less accessible and attractive than other MSC niches. Consequently, the human vertebral body as a stem cell niche is poorly studied: It is not only unclear whether dysfunction of MSCs contributes to the pathogenesis of osteoporosis; even a simple fundamental characterization of MSCs from vertebral bodies is nonexistent.

Therefore, the current study aimed to isolate MSCs from vertebral bodies of osteoporotic and non-osteoporotic control patients, to characterize both MSC groups, and to investigate their osteogenic differentiation activity using different approaches.

## 2. Results

### 2.1. Morphology and Proliferation Rate of BMSCs from Osteoporotic and Non-Osteoporotic Control Donors

**Figure 1:**
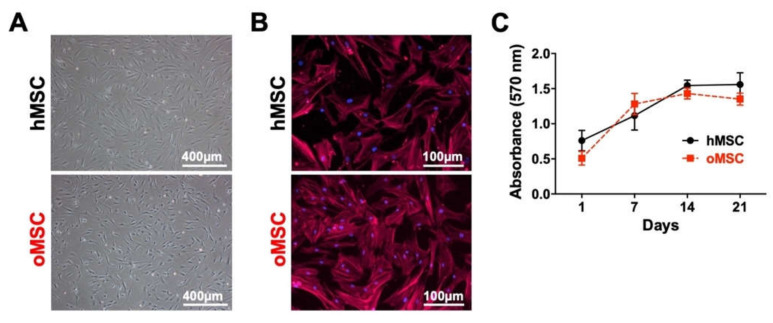
Mesenchymal bone marrow-derived stromal cells (BMSC) from vertebral bodies’ bone marrow of osteoporotic donors (oMSC) and healthy control donors (hMSC) showed comparable growth behavior and morphology. (**A**) BMSCs from both groups showed typical fibroblastic morphology and comparable size at passage 1. (**B**) BMSCs at passage 3 were cultured as a monolayer and stained for cytoskeleton-actin (red) and nuclei (blue). (**C**) Growth behavior of BMSCs from both groups was assessed using MTT assay through absorbance measurement (570 nm) at indicated time points. Data are expressed as average ± SD of 5 donors per group.

Using bright-field microscopy, BMSCs from both groups showed typical bipolar spindle-shaped and fibroblast-like morphology at passage 1 (Figure 1A). They also showed a similar cell morphology after actin labeling at passage 3 (Figure 1B). We further addressed the question of whether MSCs with osteoporotic and non-osteoporotic background exert different proliferation behavior. To this end, an MTT assay was used to assess cell metabolic activity as an indirect measurement of cell proliferation by reflecting the number of viable cells. Of note, both groups showed continuous cell growth, and we did not detect any significant difference between the cellular densities of osteoporotic MSCs (oMSCs) and non-osteoporotic healthy MSCs (hMSCs) at any given time point (Figure 1C).

### 2.2. Phenotypic Analysis and Immunomodulatory Capacity

**Figure 2:**
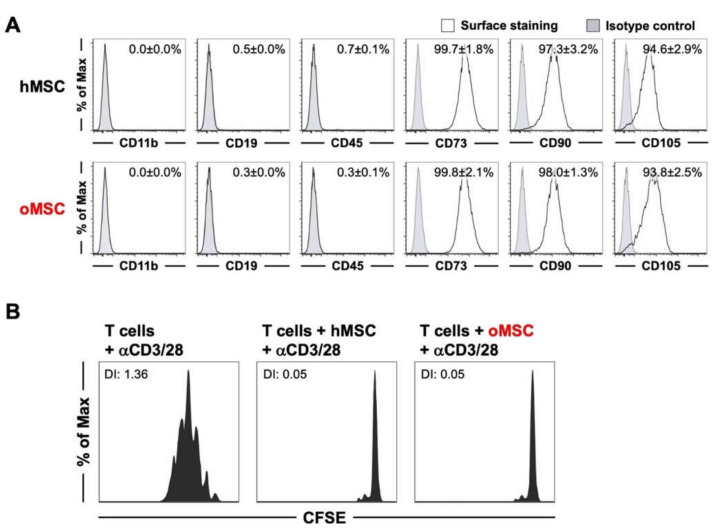
Mesenchymal stromal cells (MSC) of healthy control donors (hMSC) and osteoporotic donors (oMSC) exhibited comparable surface marker expression and immune-modulatory capacity. (**A**) Flow cytometric surface marker expression analysis of MSCs from osteoporotic and non-osteoporotic donors at passage 3–4. The percentage of positive cells is indicated in the top right corners. (**B**) hMSCs and oMSCs were tested for their immunomodulatory capacity by suppressing the proliferation of CD8^+^ T cells. Carboxyfluorescein succinimidyl ester (CFSE)-labeled human CD8^+^ T cells were stimulated with αCD3/28-coated beads in the absence or presence of hMSCs or oMSCs, and proliferation profiles were flow cytometrically analyzed. Division index (DI) as a measure of cell proliferation is depicted in the top left corners. Data are expressed as average ± SD of five donors per group.

A basic surface marker characterization was performed using flow cytometry to further analyze MSCs from osteoporotic and non-osteoporotic healthy controls. All MSCs were analyzed for the surface markers CD11b, CD19, CD45, CD73, CD90, and CD105 (Figure 2A).

BMSCs from both groups positively expressed the common surface markers CD73, CD90, and CD105, and were found to be negative for the CD11b, CD19, and CD45 (Figure 2A), which is in line with the criteria defined by the International Society for Cellular Therapy (ISCT) [27]. Furthermore, we did not find any significant difference between hMSCs and oMSCs in relation to their expression of common MSC surface markers.

BMSCs were analyzed for their immunomodulatory properties in accordance with the ISCT criteria. To this end, hMSCs and oMSCs were tested for their capacity to inhibit the proliferation of CD8^+^ T cells. Specifically, human CD8^+^ T cells were labeled with Carboxyfluorescein succinimidyl ester (CFSE), then stimulated with αCD3/28-coated beads in the presence or absence of MSCs from both groups, and T cell proliferation was flow cytometrically visualized by CFSE dilution after 3 days. In the presence of αCD3/28, T cells strongly proliferated, as could be seen by a CFSE proliferation profile with several peaks (Figure 2B). However, in the presence of both hMSCs and oMSCs, the proliferation of αCD3/28-activated CD8^+^ T cells was completely abolished. We did not detect any significant difference between the immunomodulatory capacity of hMSCs and oMSCs.

### 2.3. Osteogenic, Adipogenic, and Chondrogenic Differentiation

**Figure 3:**
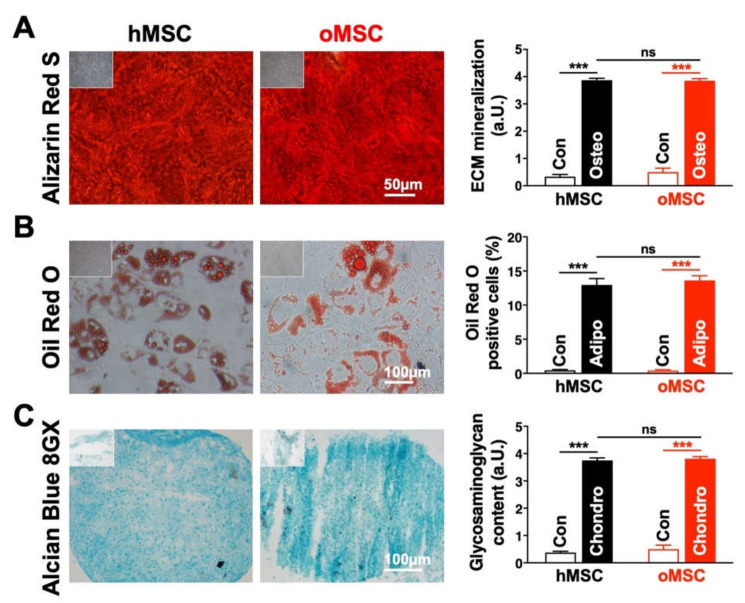
Comparable osteogenic, adipogenic, and chondrogenic differentiation potential. Mesenchymal stromal cells (MSC) of healthy control donors (hMSC) and osteoporotic donors (oMSC) at passage 3–4 were induced towards (**A**) osteoblast (Osteo), (**B**) adipocyte (Adipo), and (**C**) chondrocyte (Chondro) lineages. MSCs in culture medium without any osteogenic, adipogenic, or chondrogenic induction supplement were used as controls (inserts in the top left corners). Differentiation success was confirmed via (**A**) Alizarin Red S, (**B**) Oil Red O, and (**C**) Alcian Blue 8GX stains. (**A**,**C,** right) The extracellular matrix (ECM) mineralization and glycosaminoglycan content were evaluated using a semi-quantitative score based on the staining intensity and area (see Materials and Methods, 4.7 and 4.8). (**B**, right) The Oil Red O staining was determined by measuring the percentage of cells stained positive for Oil Red O using the cellSens Dimension software (see Section 4.6). The same magnification was used for all analyses. Con: control, a.U.: arbitrary unit, ns: not significant. Data are expressed as average ± SD of three donors (adipogenic and chondrogenic differentiation) and five to eight donors (osteogenic differentiation) per group. *** *p* < 0.001, Student’s *t*-test.

Next, we characterized the osteogenic and chondrogenic differentiation potential of MSCs from osteoporotic and healthy control donors. First, we induced both BMSC groups towards the osteoblast lineage, and the osteogenic differentiation was confirmed via Alizarin Red S staining (Figure 3A, left).

MSCs from both osteoporotic and non-osteoporotic donors showed strong mineralization indicating their solid osteogenic capacity. BMSC control cultures from the corresponding groups were cultured under the same conditions without any osteogenic supplement and were stained negative for Alizarin Red S (Figure 3A, left, inserts in the top left corners). A direct comparison of the mineralization of hMSCs and oMSCs revealed no difference in their mineralogenic potential, suggesting that BMSCs from osteoporotic patients were not impaired. The osteogenic differentiation was further quantified by evaluating the mineralization rate by setting a semi-quantitative score based on the intensity of Alizarin Red S staining, which confirmed the successful and comparable osteogenic potential of both groups (Figure 3A, right). The mineralogenic effect was additionally assessed during the linear phase of extracellular matrix (ECM) mineralization, at day 7 and day 14, to avoid any possible overlooking of delicate differences between hMSCs and oMSCs; however, no differences in ECM mineralization was observed (Figure S1).

In the next step, we investigated the adipogenic differentiation potential of hMSCs and oMSCs. During the adipogenic differentiation process, BMSCs from both groups accumulated significant amounts of lipid-rich vacuoles that were confirmed via Oil Red O staining, indicating the successful differentiation towards the adipocyte lineage (Figure 3B, left). Both groups generated a great number of lipid-storing cells, and we did not find any significant difference between hMSCs and oMSCs. This result was also confirmed by a quantitative evaluation of the percentage of Oil Red O positive cells (Figure 3B, right).

As the last step, we also differentiated MSCs towards the chondrocyte lineage. At the end of the chondrogenic induction period, BMSCs from both groups showed typical characteristics of glycosaminoglycan matrix that were confirmed via Alcian Blue staining. This staining demonstrated the capability of both MSC groups to differentiate towards the chondrocyte lineage (Figure 3C, left) and further uncovered that both groups differentiated to the same extent. The chondrogenic differentiation rate was further assessed by a semi-quantitative scoring, which verified the similar differentiation potential (Figure 3C, right). In summary, hMSCs and oMSCs could be shown to possess a solid multilineage differentiation potential, and at the end stage of the differentiation procedure, no differences in their differentiation potential could be observed.

### 2.4. Alkaline Phosphatase Intensity and Activity during Osteogenic Differentiation Process

**Figure 4:**
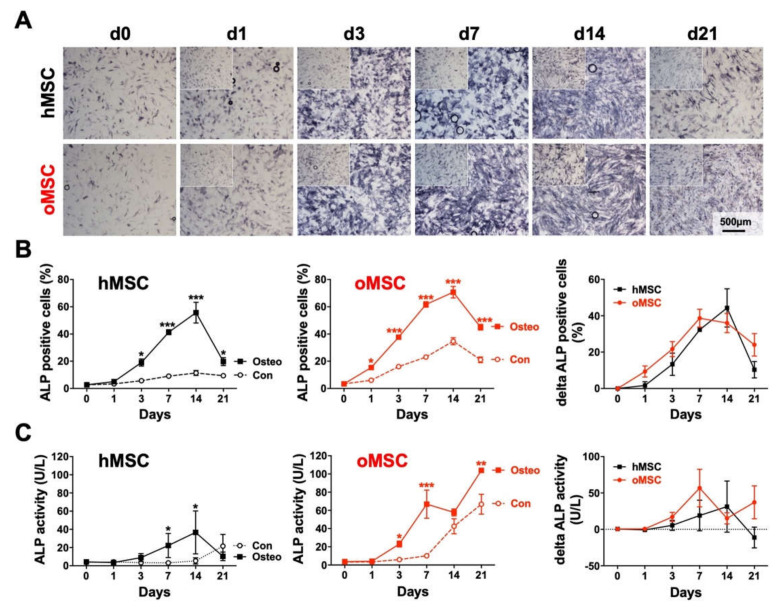
Alkaline Phosphatase (ALP) intensity and activity during the osteogenic differentiation process. (**A**) Mesenchymal stromal cells (MSC) of healthy control donors (hMSC) and osteoporotic donors (oMSC) at passage 3 were induced towards the osteoblast lineage for 21 days, and ALP staining was performed at indicated time points. A culture medium without any osteogenic induction supplement was used as control (inserts in the top left corners). The same magnification was used for all analyses. (**B**) The relative ALP staining intensity of both BMSCs was evaluated by measuring the percentage of cells stained positive using the cellSens Dimension software, and the delta of ALP positive cells was determined by subtracting the non-induced controls from the induced MSCs. (**C**) ALP activity of hMSCs and oMSCs was determined with the help of 4-Methylumbelliferyl phosphate disodium salt substrate using a fluorometric assay at indicated time points. The delta ALP activity was determined by subtracting non-induced from induced MSCs. Data are expressed as average ± SD of three to eight donors per group. * *p* < 0.05, ** *p* < 0.01, *** *p* < 0.001, one-way ANOVA.

To further analyze the osteogenic differentiation potential of both groups, hMSCs and oMSCs were induced towards the osteoblast lineage and stained for alkaline phosphatase (ALP) at different time points during the differentiation process (Figure 4A, Figure S2). In comparison to the corresponding controls, the ALP staining indicated a steady increase in the ALP intensity in both MSC groups.

The ALP staining of MSCs from both groups was further evaluated by measuring the percentage of cells stained positive for ALP. Interestingly, both hMSCs and oMSCs showed a comparable steady increase in the percentage of cells stained positive for ALP, reaching their peak at day 14 (Figure 4B). oMSCs seemingly represented a greater proportion of ALP positive cells; however, the control group of oMSCs also presented more ALP positivity, suggesting that MSCs from osteoporotic patients might exhibit higher ALP activity. When considering the real osteogenic potential, which is reflected by the difference between induced and non-induced MSCs, both hMSCs and oMSCs did not show any significant difference (Figure 4B, right). Interestingly, the percentage of cells positive for ALP decreased from day 14 to day 21 in both groups (Figure 4B).

In parallel to the ALP staining, we also assessed the ALP activity using the same experimental setting (Figure 4C). At day 3 of induction, BMSCs from both groups already showed an increased ALP activity compared to their corresponding controls. When induced towards the osteogenic differentiation, oMSCs showed a stronger increase in ALP activity than hMSCs at all time points. However, when normalized to the control samples, no significant differences could be seen between both groups. The ALP activity peak was reached at day 7 for oMSCs and at day 14 for hMSCs, but differences were not significant.

### 2.5. Assessment of Osteogenic Differentiation

**Figure 5:**
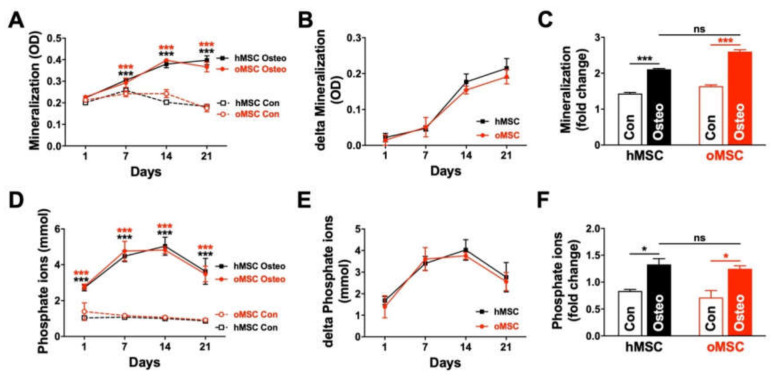
Comparable mineralization and phosphate ion release during the osteogenic differentiation process. Mesenchymal stromal cells (MSC) from osteoporotic (oMSC) and healthy non-osteoporotic donors (hMSC) at passage 3–4 were induced towards the osteoblast lineage for 21 days. Culture medium without any osteogenic induction supplement was used as control. (**A**) The mineralization process of both groups was assessed by optical density (OD) measurement at the indicated time points, and (**B**) the delta mineralization rate was determined by subtracting the non-induced controls from the induced MSCs. (**C**) The overall mineralization fold change was calculated using the ratio day 21/day 1. (**D**) The osteogenic differentiation process of MSCs from both groups was assessed by measuring the inorganic free phosphate ion release into the cell culture supernatant at the indicated time points, and (**E**) the delta phosphate ions release was determined by subtracting the values of non-induced controls from the induced MSCs. (**F**) The fold change of the total phosphate ion release from both MSC groups was determined using the ratio day 21/day 1. ns: not significant. Data are expressed as average ± SD of three to five donors per group. * *p* < 0.05, *** *p* < 0.001, one-way ANOVA.

The mineralization process was further assessed through mineral deposition and phosphate ion release, as described previously [22]. hMSCs and oMSCs were induced to differentiate towards the osteoblast lineage. Cell culture medium without any osteogenic induction supplement was used as control. Mineralization of BMSCs was evaluated by optical density (OD) measurements of MSC monolayer cultures at different time points during the osteogenic differentiation period, thereby quantifying the deposition of inorganic crystals (Figure 5A).

Both induced MSC groups showed a continuous increase in crystal deposition (OD) over time compared to the corresponding non-induced controls. Induced MSCs from osteoporotic and healthy patients indicated a similar tendency at all time points. A minor decrease in OD values in the controls was observed between day 7 to day 21 in both hMSCs and oMSCs (Figure 5A). To better visualize the absolute increase in the mineralization, delta values between induced and non-induced samples were calculated, which confirmed the continuous mineralization increase (Figure 5B). This was further confirmed by calculating the fold change of the OD shift over time (Figure 5C).

In addition to the OD measurements, the osteogenic differentiation process was also monitored through the determination of inorganic free phosphate ion release into the supernatant at different time points. BMSCs from both groups, osteoporotic and non-osteoporotic, demonstrated a comparable phosphate ion release at all time points during the osteogenic differentiation (Figure 5D+E). In general, phosphate ion release peaked at day 14. The phosphate ion level decreased until day 21 but was still elevated in comparison to the osteogenic initiation (day 1), which was further confirmed by the overall fold change of the phosphate ion release (Figure 5F).

### 2.6. Osteoblast Marker Gene Expression

Finally, the osteogenic differentiation of BMSCs from both groups was assessed using RT-PCR by investigating the relative mRNA expression of *ALPL*, *COL1A1*, *RUNX2,* and *SOX9* at different time points.

**Figure 6:**
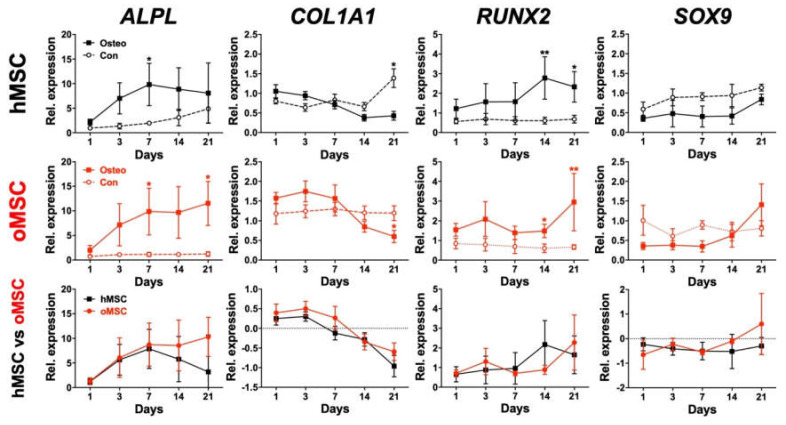
Expression dynamics of common osteogenic gene markers. Mesenchymal stromal cells (MSC) from osteoporotic (oMSC) and non-osteoporotic healthy control donors (hMSC) at passage 3–5 were induced towards the osteoblast lineage for 21 days. Culturing medium without any osteogenic induction supplement was used as control. The relative mRNA expression of *ALPL*, *COL1A1*, *RUNX2,* and *SOX9* was investigated at the indicated time points during the osteogenic differentiation. Data analysis was performed using ddCT values normalized to *GAPDH*. Data are expressed as average ± SD of three to four donors per group. For a direct comparison of the hMSC vs. oMSC groups (bottom panels), non-induced samples were subtracted from induced MSCs to determine the delta expression level. * *p* < 0.05, ** *p* < 0.01, Mann–Whitney U.

The early osteoblast marker *ALPL* showed a continuous increase from day 1 to day 7 in both groups. From day 7 to day 21, hMSCs showed decreased *ALPL* expression, whereas oMSCs slightly, but not significantly, increased gene expression from day 7 to day 21 (Figure 6). The osteoblast lineage-specific gene, *COL1A1*, showed comparable expression during the whole osteogenic differentiation process and decreased from day 7 to day 21 in both groups (Figure 6). *RUNX2* was slightly upregulated at the end of the osteogenic differentiation process in both groups (Figure 6). *SOX9*, which is a negative osteogenic marker [28,29,30,31], was downregulated for most of the differentiation period. oMSCs showed a slight but not significant increase in *SOX9* expression at day 21 (Figure 6). In summary, hMSCs and oMSCs presented a similar gene expression dynamic, and no significant differences could be detected between both groups.

## 3. Discussion

The vertebral body as a stem cell niche is, at most, sparsely described, and it is unclear whether dysfunction of MSCs contributes to the pathogenesis of osteoporosis. Therefore, the current study aimed to isolate and characterize MSCs from the lumbar spine vertebral body of non-osteoporotic and osteoporotic patients. MSCs from both groups fulfilled the minimal MSC criteria in line with the ISCT guidelines [27]. They demonstrated fibroblast-like morphology, similar proliferation tendencies, typical MSC surface markers, immunomodulatory capacity, and comparable trilineage potential. Interestingly, none of the parameters used to investigate vertebral body-derived MSCs from osteoporotic and healthy patients demonstrated significant differences between these two groups. This is in contrast to recently reported studies that showed an increased formation of adipocytes and a reduced production of osteoblastic cells [32]. In a later study, it has been shown that muscle-derived MSCs are less deficient than femur head-derived MSCs from osteoporotic patients compared to controls, indicating that the MSC niche must be taken into consideration [33].

Most importantly, in our study, a close investigation of the osteogenic differentiation potential indicated that MSCs from osteoporotic patients were not impaired when compared to MSCs from non-osteoporotic patients. The obtained results confirmed that both hMSCs and oMSCs exhibited a potent capacity to differentiate towards the osteoblastic lineage in vitro, reflected by a steadily intensifying ALP staining from day 1 to day 14. We also noticed a decrease in ALP staining from day 14 to day 21. This effect, however, is not specific for vertebral MSCs. It has been reported before for other MSCs, but no reasonable explanation for this decline was given [34]. Indeed, ALP is an early marker of osteoblastic differentiation, whereas ECM mineralization is associated with late osteoblastic differentiation and transition towards osteocytes [35,36]. Interestingly, the relative mRNA of *ALPL* has been reported to be decreased at day 21 and day 28 in MSCs under osteogenic differentiation [37]. We also noticed a stagnation of ALP expression, which may explain the decrease in ALP staining at day 21 in our current study in both hMSCs and oMSCs. Further, the dynamic transition from osteoblasts to osteocytes should also be taken into consideration. It has been reported that primary osteoblasts from mice under osteogenic differentiation expressed osteocyte markers and showed decreased *ALPL* expression [38]. Therefore, the decrease in ALP staining at day 21 could also be due to the transition of osteoblasts to osteocytes, but this remains to be clarified.

Furthermore, we noticed a slight increase in ALP activity in osteogenically induced oMSCs and their corresponding control compared to hMSCs (Figure 4C). However, this effect did not reflect ECM mineralization determined via OD measurement, where no difference was observed (Figure 5B). It has been reported in a comparative analysis using different cells, including BMSCs and a variety of osteogenic and mineralizing media conditions, that ALP activity is not proportional to mineralization levels [39]. It has been shown that ALP activity increases in confluent monolayer MSCs during the first three weeks of differentiation [39], and in some cases, MSC cultures can produce high levels of ALP in vitro which do not fully correlate with the extent of mineralization [40].

Alizarin Red S has been traditionally used as the golden standard to evaluate and quantify ECM mineralization in vitro [41]. Nevertheless, this method presents a number of disadvantages, including culture disruption for fixation, preventing further measurements [42]. In our current study, therefore, we made use of alternative refined assays to quantitatively follow up the mineralization process continuously and accurately. To this end, we employed a methodology to analyze the mineralization process by monitoring crystal deposits by measuring the OD of monolayer cultures of hMSCs and oMSCs during the osteogenic differentiation, as reported previously [37]. In previous studies, the OD was found to correlate with Alizarin Red S quantification, which was further supported by phosphate ion release in our current study [37].

The measured OD of the deposited crystals did not indicate any significant difference between hMSCs and oMSCs; however, this does not exclude potential differences in crystal composition. A qualitative analysis of the deposited crystals to determine their composition and crystal types should be investigated further in the future. It has been shown that cultured BMSCs on collagen I/III gel led to hydroxyapatite/calcium crystal deposition, as well as ECM proteins, in a similar manner to functional osteocytes and osteoblasts [43]. An accurate analysis to compare the chemical composition and structural properties of the deposited crystals in hMSCs and oMSCs would be of great interest as it would give deeper insights regarding the process of osteogenesis mediated by MSCs from healthy versus osteoporotic donors.

Further, we assessed the osteogenic differentiation of hMSCs and oMSCs by monitoring the gene expression of early osteogenic markers, such as *ALPL*, *COL1A1,* and *RUNX2,* utilizing RT-PCR at different time points. It has been previously reported that the relative mRNA expression of *ALPL* was elevated at day 7 with its peak at day 14, followed by a decrease at day 21 [37,44]. Our data indicated a steady increase in *ALPL* from day 1, with its peak at around day 7 for both hMSCs and oMSCs. The observed slight decrease in the relative mRNA expression of *ALPL* in hMSCs and unchanged mRNA levels in oMSCs at day 14 may explain the slight decrease in ALP staining at day 21. Additionally, the determined relative mRNA expression of *COL1A1* showed a comparable increase already at day 1 to day 7, and then a shift was observed showing a gradual downregulation towards day 21 in both hMSCs and oMSCs compared to their corresponding controls. Similar findings have been reported previously by assessing *COL1A1* gene expression during osteogenic differentiation of MSCs from healthy patients [34,37,45]. The transcription factor *RUNX2* plays a major role in osteoblast differentiation and bone formation and was shown to be expressed at a relatively similar level during in vitro differentiation of primary human osteoblasts [46,47,48,49]. In accordance with the previously reported findings, the expression of *RUNX2* was found to be increased and comparable at all time points in both hMSCs and oMSCs compared to their corresponding controls.

The transcription factor *SOX9* is known to play a key role in chondrogenesis and endochondral bone formation [50,51] and has been shown to be a major regulator in direct osteogenesis by directly interacting with *RUNX2* [29,51]. It has been reported that *SOX9* mRNA expression was higher in the control medium compared to MSCs under osteogenic differentiation on days 2, 7, and 14, but not at day 21 [29]. Our current data indicated a clear downregulation of *SOX9* mRNA expression already at day 1 until day 14 in both hMSCs and oMSCs compared to their corresponding undifferentiated controls. Loebel et al. showed the impact of *SOX9* downregulation in mineralization of human MSCs in vitro, demonstrating that *SOX9* plays a major role in regulating direct osteogenesis. Moreover, the *RUNX2*/*SOX9* ratio has been proposed as an early indicator for osteoblastic differentiation of human MSCs in vitro [29]. Further studies are required to assess the expression of *SOX9* and its relation with *RUNX2* in the future. The comparable expression of *ALPL*, *COL1A1*, *RUNX2,* and *SOX9* in both hMSCs and oMSCs additionally supports the similarity of their osteogenic differentiation potential in vitro. Taken together, the current study presents similar differentiation properties of vertebral bone marrow-derived mesenchymal stromal cells from osteoporotic and healthy patients in vitro using different approaches.

This finding is in contrast to studies from other niches, which were performed in animal models for osteoporosis and osteoporotic patients that showed a reduced MSC proliferation rate in osteoporotic patients and, most importantly, an impaired osteogenic differentiation potential [10,11,12,52]. Taken together, these findings neatly show how diverse MSCs from different niches are and how important it is to investigate tissue source-specific differences. Recent studies have already demonstrated that MSCs derived from vertebrae can be maintained in vitro for a greater number of steps [53]. They further showed that MSCs from vertebral bodies were able to differentiate even more efficiently into all mesenchymal lineages under osteogenic, adipogenic, and chondrogenic conditions. Another study demonstrated that vertebral body MSCs possessed a comparable phenotype and proliferative capacity but higher chondrogenic and osteogenic properties than MSCs from the iliac crest [54]. Basically, these studies demonstrate the superiority of vertebral MSCs in terms of their osteogenic differentiation behavior. One could argue that vertebral MSCs indicate above-average osteogenic differentiation behavior under homeostatic conditions, which is highly plausible considering their anatomic location. Interestingly, in our study, we found that vertebral body MSCs from osteoporotic patients have similar proliferation and differentiation capability in comparison to MSCs from healthy control donors, which is in contrast to previous reports from other MSCs niches, such as femur head, iliac crest, and muscle [11,32,33].

Obviously, a remaining question is why vertebral MSCs are so different in comparison to MSCs from other niches. One explanation could lie in the local microenvironment of the vertebral body bone marrow, which could shape the fate of local MSCs. This local influence could be the cellular composition that interferes with MSCs, or it could be a simple molecular trigger. A recent publication identified the histone methyltransferase enhancer of Zeste homology 2 (EZH2), which regulates the lineage commitment of MSCs and, therefore, contributes to the pathology of osteoporosis [55]. Although more and more molecular mechanisms have been identified, we are still at the beginning of understanding the fate determination of abnormal versus normal BMSCs. However, focusing on MSCs cannot be the only solution to treat osteoporosis, as our in vitro data suggest that vertebral body osteoporosis may not primarily be due to abnormal osteogenic properties of local MSCs. Certainly, ex vivo or in vivo data will be needed to formally prove this hypothesis in a more physiological context, as in vitro expanded MSCs potentially possess a different phenotype.

In summary, this study characterized MSCs from the lumbar spine vertebral body of non-osteoporotic and osteoporotic patients and found that vertebral body MSCs from osteoporotic patients were not impaired, but they rather possessed full osteogenic potential compared to MSCs from non-osteoporotic patients. These results highlight the highly important influence of the tissue source and its local microenvironment for the MSC phenotype.

## 4. Materials and Methods

### 4.1. Tissue Donors and Isolation of Bone Marrow-Derived MSCs

Recruitment of human subjects for collecting bone marrow aspirate was approved by the local ethics committee (University Hospital Bonn, project ID: 102/10, approval date: 20 July 2010) and was conducted in accordance with the approved guidelines as well as the declaration of Helsinki. BMSCs were harvested from vertebral body aspirates of the lumbar spine of osteoporotic (oMSC) and non-osteoporotic healthy control donors (hMSC), which were undergoing spondylodesis and kyphoplasty procedures, respectively. All osteoporotic patients were diagnosed with grade II osteoporosis (*n* = 12) and had an average age of 69 years (8 females, 4 males). Healthy patients (*n* = 5) had an average age of 62 years (2 females, 3 males). MSCs were isolated through gradient centrifugation using Biocoll separating solution (Biochrom AG, Berlin, Germany) and their ability to adhere to tissue culture plastic, as described previously [22]. Cells were cultured and expanded in polystyrene cell culture flasks (Greiner Bio-One GmbH, Frickenhausen, Germany) using Dulbecco’s Modified Eagle Medium (DMEM) (Gibco by Life Technologies, Darmstadt, Germany) containing 10% fetal bovine serum (FBS), 1% l-glutamine, 1% penicillin–streptomycin (Biochrom AG, Berlin, Germany) under standard conditions (37 °C, 95% humidity, atmospheric O_2_ and 5% CO_2_). After isolation, BMSCs were expanded via subculturing for two passages and then stored at −150 °C until further use. All experiments reported in this study were performed using BMSCs from passage 3 to passage 5.

### 4.2. Morphologic Analysis

For morphological analysis, MSCs from osteoporotic and healthy control donors were grown to approximately 80% confluency and then fixed with 4% paraformaldehyde (PFA, pH 7) in PBS (ThermoFisher Scientific, Karlsruhe, Germany) for 5 min. After the washing step, BMSCs were permeabilized with 0.25% Triton X-100 (Sigma–Aldrich, Darmstadt, Germany) for 5 min, and an anti-actin antibody (10 µg/mL) (Abcam plc, Cambridge, UK) was applied for 10 min as well as 4′,6-Diamidino-2-phenylindole (DAPI) nucleus counterstain.

### 4.3. MTT Assay

The growth properties of hMSCs and oMSCs were indirectly measured by determining their metabolic activity using an MTT assay. To this end, cells were cultured at a density of 2 × 10^3^ cells/well in a 96-well plate as monolayer culture under standard conditions for 21 days. The culture medium was changed each third day, and the measurements were carried out at the indicated time points according to the manufacturer’s protocol using the MTT assay kit (Boster Biological Technology Co., Ltd., Pleasanton, CA, USA).

### 4.4. Flow Cytometric Analysis

Analysis of the phenotypic surface marker expression of BMSCs from osteoporotic and healthy donors was performed by flow cytometry using a BD FACS Canto II cell analyzer and FlowJo software (BD Biosciences, Heidelberg, Germany). Briefly, MSCs were resuspended in PBS with 1% FBS/2 mM ethylenediaminetetraacetic acid (EDTA) and then incubated with saturating concentrations of antibodies (ThermoFisher Scientific, Karlsruhe, Germany) for 20 min. MSCs were tested for CD11b, CD19, CD45, CD73, CD90, and CD105. Unstained cells and isotype antibodies were used as controls.

### 4.5. Immunomodulatory Capacity

Peripheral blood mononuclear cells (PBMC) were isolated out of human whole blood (*n* = 5) using a Ficoll gradient, and the resulting freshly isolated naive lymphocytes were enriched for CD8^+^ T cells using human CD8 MicroBeads (Miltenyi, Bergisch-Gladbach, Germany). Naive CD8^+^ T cells were labeled with Carboxyfluorescein succinimidyl ester (CFSE) (Molecular Probes, Leiden, Netherlands) and then washed with PBS with 1% FBS to remove extracellular CFSE. Four times ten to the fourth hMSCs or oMSCs per 24-well were cultured for 48 h to reach confluency, and then 1 × 10^6^ CD8^+^ T cells and αCD3/38-coated beads (ThermoFisher Scientific, Karlsruhe, Germany) were added. The proliferation of the CD8^+^ T cells was flow cytometrically assessed by analyzing the CFSE dilution after 3 days, as described previously [56,57].

### 4.6. Adipogenic Differentiation

MSCs with a cell density of 1 × 10^4^ cells/cm^2^ from osteoporotic and healthy donors were differentiated towards the adipocyte lineage by adding 1 µM dexamethasone, 1 µM insulin, and 200 µM indomethacin (Sigma–Aldrich, Darmstadt, Germany) to the cell culture medium, as described previously [13,22]. MSCs cultured in an unsupplemented medium were used as undifferentiated cell controls. After 21 days, cells were washed with Dulbecco’s phosphate-buffered saline (DPBS), fixed in 4% formalin (pH 7) (Carl Roth GmbH, Karlsruhe, Germany) at 37 °C for 30 min and then stained with 0.1% Oil Red O staining (Sigma–Aldrich, Darmstadt, Germany) for 30 min. The staining solution was removed, samples were kept in PBS, and pictures of several high-power fields were taken with a light microscope within 30 min. The adipogenic differentiation rate was evaluated by analyzing the captured images and quantifying the percentage of cells stained positive for Oil Red O using the cellSens Dimension software (Olympus Corporation, Hamburg, Germany), as described previously [22].

### 4.7. Chondrogenic Differentiation

Differentiation of BMSCs towards the chondrocyte lineage was performed as described previously [13,22]. In detail, three dimensional (3D) pellets consisting of 2.5 × 10^5^ cells were resuspended in a culture medium and centrifuged at 500× *g* in a 15 mL conical tube. Pellets were cultivated in chondrogenic medium with loosened cap under standard conditions (37 °C, 95% humidity, atmospheric O_2,_ and 5% CO_2_) for 21 days using high-glucose DMEM medium supplemented with 1 µg/mL insulin, 1 ng/mL transferrin, and 1 ng/mL sodium selenite, 0.1 µM dexamethasone, 50 µM 2-phosphate-l-ascorbic acid trisodium salt, and 10 ng/mL transforming growth factor beta-1 (Sigma–Aldrich, Darmstadt, Germany). MSCs cultured in an unsupplemented medium were used as undifferentiated cell controls. 3D pellets were fixed with 4% PFA (pH 7), cut into 12 μm cryosections, and stained with Alcian Blue dye (1% *w*/*v* Alcian blue 8GX, in 3% acetic acid solution, containing 0.1 M CaCl_2_, pH 1) (Sigma–Aldrich, Darmstadt, Germany). After staining, a selection of images was taken of all cryosections from the pellet cultures, and the glycosaminoglycan content was analyzed using a semi-quantitative score based on the intensity of Alcian Blue staining, as reported before [22,58]. Undifferentiated cells served as control. The Alcian Blue staining-based scoring scale was as following: (0) negative, (1) weakly positive, (2) moderately positive, (3) markedly positive, or (4) strongly positive.

### 4.8. Osteogenic Differentiation

BMSCs from osteoporotic and healthy donors were seeded at a density of 1 × 10^4^ cells/cm^2^ and induced towards the osteoblast lineage by using a culture medium supplemented with 0.1 µM dexamethasone, 10 mM β-glycerophosphate disodium salt hydrate, and 50 µM 2-phosphate-l-ascorbic acid trisodium salt (Sigma–Aldrich, Darmstadt, Germany). A culture medium without any osteogenic induction supplement was used as control. After 7, 14, and 21 days, differentiated cells were fixed with 4% formalin (in PBS, pH 7) (Carl Roth GmbH, Karlsruhe, Germany) and stained with 40 mM Alizarin Red S (pH 4.2) (Sigma–Aldrich, Darmstadt, Germany). The ECM mineralization was determined using a semi-quantitative score based on the intensity of Alizarin Red S staining of images taken from different high-power fields, as described before [22,34]. Scoring scale: (0) negative, (1) weakly positive, (2) moderately positive, (3) markedly positive, (4) strongly positive.

### 4.9. Alkaline Phosphatase Measurement, Optical Density Measurement, and Free Phosphate Assay

MSCs from both groups were induced towards the osteoblast lineage using a cell density of 10^4^ cells/cm^2^ in 96-well plates. A culture medium without any osteogenic induction supplement was used as control. The differentiation process was investigated through different approaches.

At different time points during the osteogenic induction, BMSCs were stained with ALP (Dako, Hamburg, Germany), and the relative ALP staining intensity was evaluated by analyzing the percentage of cells stained positive for ALP using the cellSens Dimension software (Olympus Corporation, Hamburg, Germany).

ALP activity was determined through 4-Methylumbelliferyl phosphate disodium salt substrate using a fluorometric assay kit (BioVision Inc., Milpitas, CA, USA). The resulting absorbance was measured at 360 nm using a microplate reader (TECAN, Magellan, Switzerland) according to the manufacturer’s instructions.

The mineralization process was further assessed by optical density (OD) measurements at 450 nm (TECAN, Magellan, Switzerland) adapted from Loebel et al. [37]. The OD absorbance was used to assess the mineralization process during the osteogenic differentiation at different time points of the same monolayer cultures. The collected OD values were corrected by subtracting the measured values of the corresponding culture medium and osteogenic induction medium without cells. After each OD measurement, supernatants were collected, and fresh corresponding medium was added to cultures during the differentiation period.

Inorganic phosphate ion release was measured in cell culture supernatants, including media without cells, at the indicated time points using the Malachite Green Phosphate Assay Kit according to the manufacturer’s instructions (Sigma–Aldrich, Darmstadt, Germany) [59].

### 4.10. Real-Time Polymerase Chain Reaction

To analyze the gene expression of common osteoblast markers, hMSCs and oMSCs were induced towards the osteoblast lineage, and real-time polymerase chain reaction (RT-PCR) was performed, as described previously [22]. Briefly, TRIzol reagent (Ambion, Life Technologies, Darmstadt, Germany) and chloroform:isoamyl alcohol (24:1) (PanReac AppliChem, Darmstadt, Germany) were used for mRNA extraction. Then, 1 µg mRNA was reverse transcribed using a Transcriptor First Strand cDNA Synthesis Kit (Roche Diagnostics GmbH, Mannheim, Germany), and RT-PCR was conducted using LightCycler 480 SYBR Green I Master according to the manufacturer’s instructions (Roche Diagnostics GmbH). Amplifications ran at 95 °C for denaturation, 60 °C for primer annealing, and 72 °C for primer extension 10 s each for 45 cycles. Primer sequences are listed in Table 1. Data analysis was performed using the ddCT method [60] determined by normalization to *GAPDH* [44].

### 4.11. Statistics

Data are expressed as average ± SD of 3–8 biological replicates (donors per group) as indicated. Statistical analysis was carried out using GraphPad Prism 7 (GraphPad, La Jolla, CA, USA). The Shapiro–Wilk test was used to test for normal distribution. For data with Gaussian distribution, a two-tailed Student’s *t*-test or one-way analysis of variance (ANOVA) were used. For non-Gaussian distributed data, the Mann–Whitney U testing was used. Significance levels are marked as * *p* < 0.05, ** *p* < 0.01, *** *p* < 0.001.

## Figures and Tables

**Table 1:** Real-Time Polymerase Chain Reaction (RT-PCR). Accession numbers, size of the products, and primer sequences used for determining the relative gene expression of *ALPL*, *COL1A1*, *RUNX2,* and *SOX9* in mesenchymal stromal cells (MSCs) during osteogenic differentiation.

Gene	Primer Sequence	Product Length	Accession Number
*GAPDH*	fwd: 5′CTCTGCTCCTCCTGTTCGAC3′ rev: 5′ACCAAATCCGTTGACTCCGA3‘	109 bp	NM_002046.5
*ALPL*	fwd: 5′TTTATAAGGCGGCGGGGGTG3′ rev: 5′AGCCCAGAGATGCAATCGAC3′	198 bp	NM_000478.5
*COL1A1*	fwd: 5′TGCTCGTGGAAATGATGGTG3′ rev: 5′CCTCGCTTTCCTTCCTCTCC3′	449 bp	NM_000088.3
*RUNX2*	fwd: 5′GCGCATTCCTCATCCCAGTA3′ rev: 5′GGCTCAGGTAGGAGGGGTAA3′	176 bp	NM_001024630.3
*SOX9*	fwd:5′AGGAAGTCGGTGAAGAACGG3′ rev: 5′AAGTCGATAGGGGGCTGTCT3′	275 bp	NM_000346.3

## Supplementary Materials

Supplementary materials can be found at https://www.mdpi.com/1422-0067/21/21/8309/s1. Figure S1: Mineralization of extracellular matrix (ECM) during the osteogenic differentiation process of mesenchymal stromal cells of healthy (hMSC) and osteoporotic (oMSC) donors visualized via Alizarin Red S staining at day 7, 14, and 21; Figure S2: Control samples for alkaline phosphatase (ALP) staining during the osteogenic differentiation process of mesenchymal stromal cells of healthy (hMSC) and osteoporotic (oMSC) donors at day 0, 1, 3, 7, 14, and 21.

## Abbreviations

ALP	Alkaline phosphatase
*ALPL*	Alkaline phosphatase gene
BMSC	Bone marrow-derived mesenchymal stromal cells
CD	Cluster of differentiation
cDNA	Complementary deoxyribonucleic acid
CFSE	Carboxyfluorescein succinimidyl ester
*COL1A1*	Collagen, type I, alpha 1
DAPI	4′,6-Diamidino-2-phenylindole
ddCT	Delta-delta-Ct
DMEM	Dulbecco’s modified Eagle’s medium
DPBS	Dulbecco’s phosphate-buffered saline
ECM	Extracellular matrix
EDTA	Ethylenediaminetetraacetic acid
FBS	Fetal bovine serum
*GAPDH*	Glyceraldehyde-3-phosphate dehydrogenase
hMSC	Healthy mesenchymal stromal cells
ISCT	International Society for Cellular Therapy
mRNA	Messenger ribonucleic acid
MSC	Mesenchymal stromal cells
OD	Optical density
oMSC	Osteoporotic mesenchymal stromal cells
PBMC	Peripheral blood mononuclear cell
PBS	Phosphate-buffered saline
PFA	Paraformaldehyde
RT-PCR	Real-time polymerase chain reaction
*RUNX2*	Runt-related transcription factor 2
*SOX9*	SRY-Box Transcription Factor 9
