# Supplementary material for: Vertebral Bone Marrow-Derived Mesenchymal Stromal Cells from Osteoporotic and Healthy Patients Possess Similar Differentiation Properties In Vitro
Source: Int J Mol Sci. 2020 Nov 5;21(21):8309. doi: 10.3390/ijms21218309 (PMC7663957; doi:10.3390/ijms21218309)
Supplement: Supplementary file 1 [file ijms-21-08309-s001.pdf]

International Journal of *Molecular Sciences*

Supplementary Materials

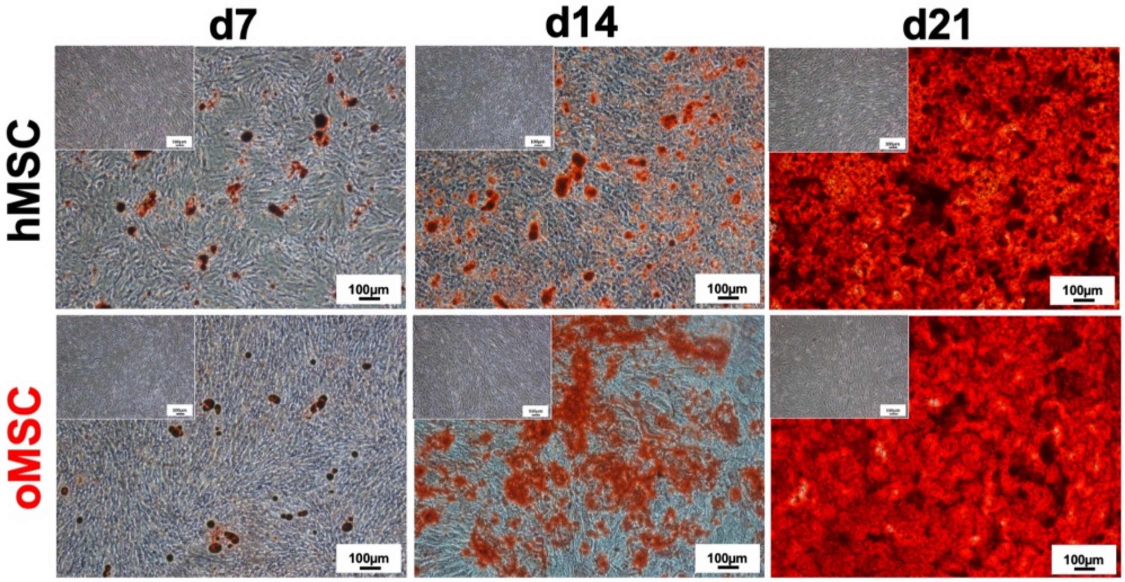

Figure S1. Mineralization of extracellular matrix (ECM) during the osteogenic differentiation process of mesenchymal stromal cells of healthy (hMSC) and osteoporotic (oMSC) donors (p3). Alizarin Red S staining was used to visualize ECM mineralization at indicated time points (d7, d14, d21). MSCs in culture medium without any osteogenic supplement were used as controls (inserts in top left corners).

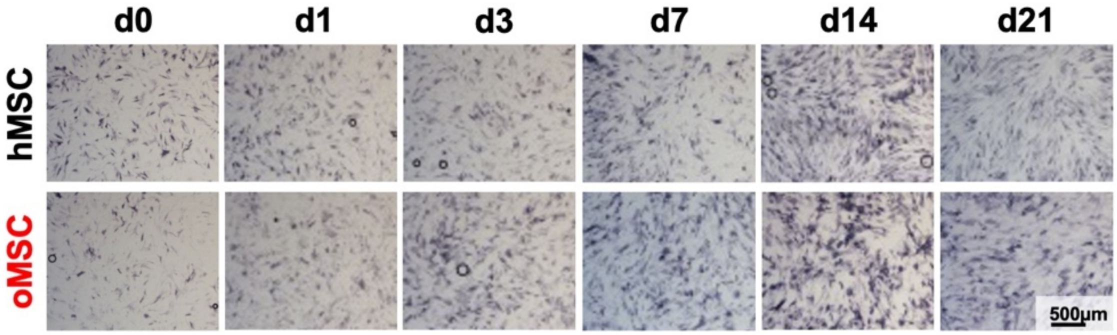

Figure S2. Control samples for alkaline phosphatase (ALP) staining. Mesenchymal stromal cells of healthy (hMSC) and osteoporotic (oMSC) donors (p3) were cultured in medium without any osteogenic induction supplement for 21 days and ALP staining was performed at indicated time points. Same magnification was used for all analyses.
